# Tibial hydatidosis: a case report

**DOI:** 10.1186/1756-0500-7-631

**Published:** 2014-09-11

**Authors:** Berhe Gebreseslassie Kassa, Melisachew Mulatu Yeshi, Amanuel Haile Abraha, Tewelde Tesfaye Gebremariam

**Affiliations:** Orthopedic Surgery Unit, School of Medicine, College of Health Sciences, Mekelle University, Mekelle, Ethiopia; Department of Pathology, School of Medicine, College of Health Sciences, Mekelle University, Mekelle, Ethiopia; Department of Internal Medicine, School of Medicine, College of Health Sciences, Mekelle University, Mekelle, Ethiopia; Department of Medical Microbiology and Immunology, Institute of Biomedical Sciences, College of Health Sciences, Mekelle University, Mekelle, Ethiopia

**Keywords:** Hydatidosis, Tibia, Surgery

## Abstract

**Background:**

Hydatidosis is a tapeworm infection caused by the larval stage of *Echinococcus* species*.* The organs most frequently affected are the liver and the lungs. Primary involvement of the skeleton is rare. The location of hydatid cysts in the tibia is seldom described in the medical literature, and its diagnosis is challenging and often presenting with a pathologic fracture simulating benign bone cystic lesion.

**Case presentation:**

We report a 53-year-old Tigrian woman who developed hydatid disease of the tibia.

**Conclusion:**

The diagnosis of primary bone hydatid disease, especially tibial hydatidosis, is difficult and requires high index of suspicion. Hence, orthopedic surgeons should be aware of this disease. Moreover, it should be considered in preoperative differential diagnosis of destructive bone lesions especially in endemic areas.

## Background

Hydatidosis is a parasitic tapeworm infection caused by the larval stage of *Echinococcus* species*.* The most common species that affect man are *E. granulosus* and *E. multilocularis* result in cystic hydatid disease and alveolar hydatid disease, respectively. The parasite lives in the small intestine of dogs, foxes and other carnivores as definite hosts [1, 2].

The disease may result when the eggs, which are discharged with the feces of the definitive host, are accidentally swallowed by the intermediate host [1, 2]. Then, the embryos are hatched in the duodenum of the intermediate host, and they traverse the intestinal mucosa to enter the portal circulation. Once they escape the filtering action of the lungs and the liver, the embryos finally reach the general circulation to involve the brain, kidneys, bones, and other tissues [2]. Echinococcosis of the bone is extremely low as most larvae are trapped by the liver and the lungs upon release of embryo in to the portal circulation [1, 3–6]. This accounts for only 0.5 to 2.5% of all human hydatidosis [1, 3–5]. Tibial involvement occurs in up to 10% of all bone involvements [3]. Because of its unusual presentation, its diagnosis may easily be missed, unless be kept in mind [4]. We report a case of hydatidosis of the tibia in a 53-year-old woman.

## Case presentation

**Figure 1:**
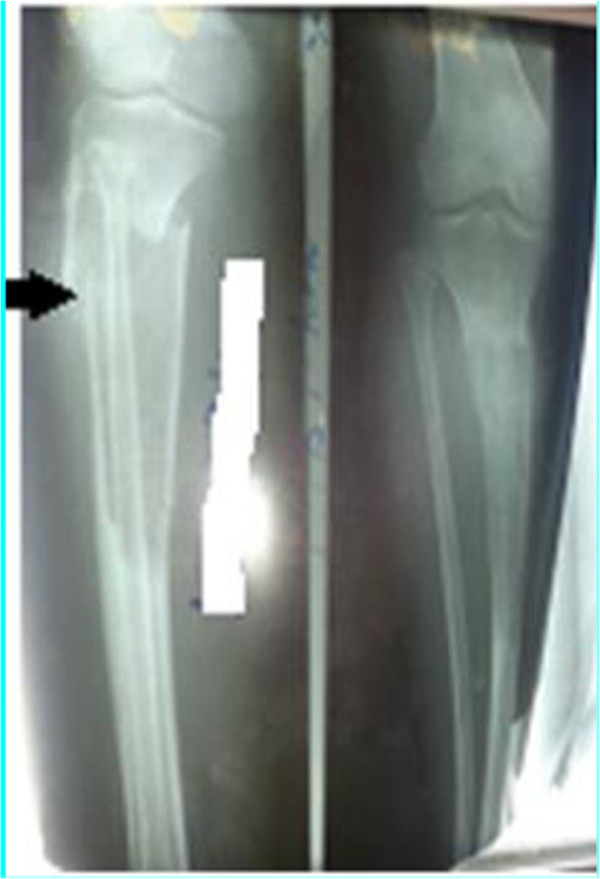
**Preoperative X-ray of the right leg (arrow).**

**Figure 2:**
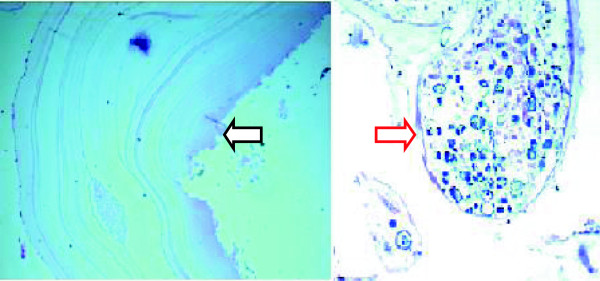
**Histopathologic examination revealing laminated acellular cyst wall with germinal layer (black arrow) and scolex (red arrow).**

**Figure 3:**
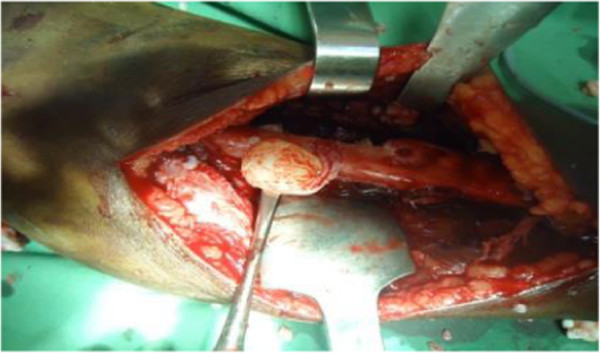
**Intraoperative procedure showing a hydatid cyst.**

**Figure 4:**
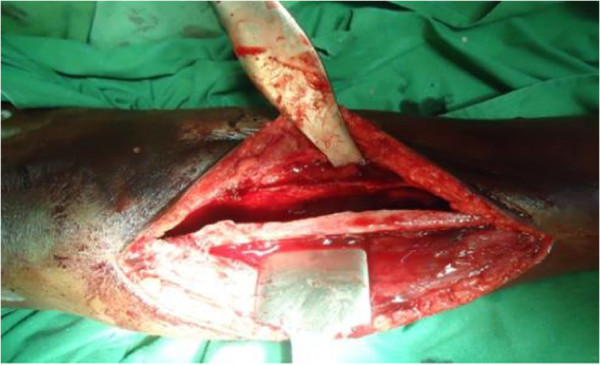
**Intramedullary tibial cavity after curettage of the cyst.**

**Figure 5:**
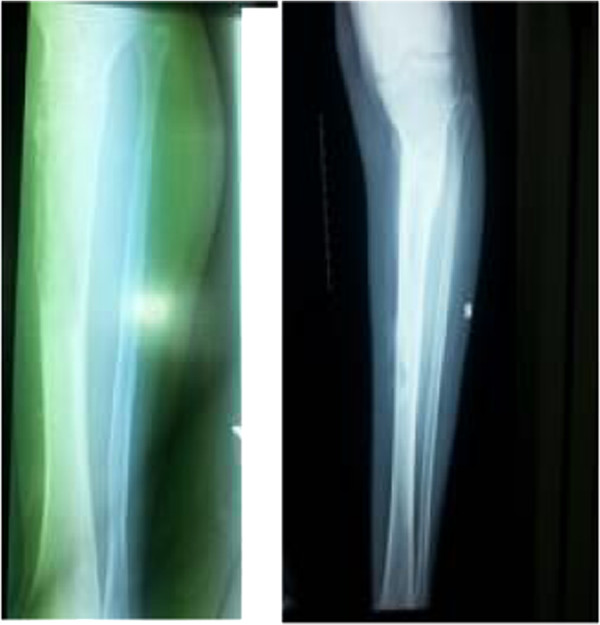
**Control x-rays of the right leg taken at four months (left) and two years (right) after definitive management.**

We present a case of 53-year-old Tigrian woman who complained from right leg pain. She had a history of taking care of animals such as sheep and dog. She had fallen from a standing height while she was walking and suffered from limping due to painful leg. She had also dull aching pain on the same leg with slight swelling for one year prior to her accident. At the first visit, she had a tumor like mass in proximal one third of the anterior part of her right leg that was painful, slightly warm, swelling with minor deformity and absence of active movement of the adjacent joints.In the primary evaluation of patient white blood cell count (WBC), differential count and erythrocyte sedimentation rate (ESR) was normal. Simple X-ray of right leg showed pathological fracture of the proximal tibia with a 13 cm oval cyst extending from the metaphysis to the diaphysis (Figure 1). Then, we took incisional biopsy of the lesion, and this was subjected to histopathologic examination and confirmed that it was a hydatid cyst (Figure 2). The patient was also screened for lung and liver involvement and her abdominal ultrasonography and chest radiography examinations were normal. Surgical resection revealed multiple membranous whitish tissues in aggregation (Figure 3). Finally, curettage of the lesion was done (Figure 4) and the patient did well and completed three cycles of treatment with Albendazole 400 mg po bid. Functional outcome was excellent, recovered motility of her right leg with no recurrence (Figure 5). She was fully weight bearing with no pain or discomfort.

## Discussion

Hydatidosis is an endemic disease in different parts of the world. The most commonly affected organs in humans are liver and lung. However, bone hydatidosis is a very rare entity accounting for 0.5 to 2.5% of total cases, as most larvae are trapped by the liver and lung upon release of embryo in to the portal circulation [1–5]. It occurs most commonly in the spine (35%) and pelvis (21%), with long bone involvement of the femur (16%) and tibia (10%) seen less commonly [3, 4, 6].

Skeletal infestation of *E. granulosus* cyst occurs by hematogenous seeding [1]. The initial location of the lesion in long bones is metaphysical or epiphyseal, later extending to the diaphysis [5]. As no connective tissue barrier form in bone, daughter cysts extend into bone infiltrating and replacing medulla, leading to the constraints of this external layer, the cysts progressively enlarge, filling the medullary cavity to a variable extent [1, 4]. This can explain why bone hydatid disease is polycystic in contrast to other non-osseous locations [1]. On the other hand, the rigid structure of the cortical bone does not allow the cyst to grow rapidly and lead to its erosion and pathological fracture and deformity, usually presents between the fourth and sixth decades of life [1, 2, 4, 7], as in the present case. This condition is rarely encountered in childhood [1, 4]. The growing cysts lead to bone destruction via three mechanisms: ischemic process through obstruction and compression of the nutrient vessels, mechanical process through compression of the surrounding tissues and a cellular process via osteoclast proliferation. There is no inflammatory reaction of bone except secondary infections which lead to sclerosis, abscess formation, and draining sinuses [8]. The cysts occasionally lie dormant in the body for up to 20 years without producing clinical signs or symptoms. The lamellated structure of the osseous tissue prevents rapid growth of the cyst along medullary and trabecular channels [1, 2, 4].

Diagnosing bone hydatid disease is more challenging and often made at an advanced stage intraoperatively, as most cases do not have a specific clinical features and imaging characteristics, and are often asymptomatic [1, 2, 5, 6, 9, 10]. Preoperative diagnosis is primarily made by imaging studies like X-ray, Computed tomography (CT) scans and Magnetic Resonance Imaging (MRI) [3–5]. X-ray findings include monolocular, bilocular, or multilocular cysts. Monolocular cysts, as in this case, are rarely observed and are characterized 87 by their oval or polycyclic nonspecific lacunae of variable sizes. Progression of the disease takes place in 2 forms: formation of diverticuli and exogenous vesiculation [5]. Although CT scan is valuable in depicting hydatid cysts, MRI is the most helpful technique, especially in the soft tissue involvement and spine [1, 6]. However, sometimes it is not possible to differentiate hydatid cyst appearance from malignancy [6]. CT scan shows well-defined single or multiple cystic lesions that may cause cortical thinning without contrast enhancement. It may also show pathological fracture, cortical destruction and soft tissue extension with calcification [1]. Accurate diagnosis may aided in some persons by eosinophilia (25 to 35% of all cases) and positive result of complement fixation tests, intradermal injection of hydatid fluid (casoni test), and indirect hemagglutination tests over a long period of time. Lesions are usually osteolytic and can involve cortical bone and extend to soft tissues [4]. Therefore, hydatid bone disease should be considered in the differential diagnosis of osteolytic lesions, especially in endemic areas. These include chronic osteomyelitis, fibrous dysplasia of bone, osteosarcoma, and benign cystic lesions. The presence of a periosteal reaction, osteosclerosis, and calcification are not specific for hydatid bone disease [5]. This case report of tibial hydatidosis is the first of its kind in Ethiopia and it was not suspected preoperatively and the immunological tests were not performed, although they are recommended.

The treatment of choice for osseous hydatid cyst is surgery with or without chemotherapy using Albendazole or Mebendazole [1, 5, 8, 11]. High rates of postoperative recurrence have highlighted the importance of adjuvant antihelmintic therapy which should be given preoperatively, if the diagnosis is known, and postoperatively in order to control the disease locally, avoid systemic spread and prevent recurrence [3, 8, 9]. Albendazole is the antiparasitic drug of choice because of its good intestinal absorption and effectiveness. It is given at a dose of 15 110 mg/kg daily in courses of 28 days. One course is given preoperatively and six or more courses postoperatively [1, 12]. Some authors advocate continuous treatment without a break, especially for complicated lesions such as hydatid disease of the bone is recommended [1, 13]. A treatment period of 2 years may be necessary although sometimes lifelong treatment is recommended. If albendazole alone is not effective, praziquantel can be added. This regimen can be used for patients with multiple recurrences and those unsuitable for surgery [1]. Our patient was managed with initial chemotherapy followed by extended curettage. Functional outcome was excellent, and she was fully weight bearing with no pain or discomfort and regained motility of her right leg with no evidence of recurrence.

## Conclusion

The diagnosis of primary bone hydatid disease is difficult and requires high index of suspicion. Therefore, orthopaedic surgeons should be aware of this disease. Moreover, osseous hydatidosis should be considered in preoperative differential diagnosis of destructive bone lesions especially in endemic areas.

## Consent

Written informed consent was obtained from the patient for publication of this Case Report and any accompanying images. A copy of the written consent is available for review by the Editor in-Chief of this journal.

## Abbreviations

WBC: White blood cells
ESR: Erythrocyte sedimentation rate
CT scan: Computed tomography scan
MRI: Magnetic resonance imaging.
